# Statistical estimation of fatal and serious injuries saved by iRAP protocols in 74 countries

**DOI:** 10.1371/journal.pone.0301993

**Published:** 2024-04-16

**Authors:** Qingfeng Li, James Bradford, Abdulgafoor M. Bachani

**Affiliations:** 1 Department of International Health, Johns Hopkins Bloomberg School of Public Health, Baltimore, MD, United States of America; 2 International Road Assessment Programme, Bracknell, Berkshire, United Kingdom; Tsinghua University, CHINA

## Abstract

**Objective:**

Road traffic crashes cause 1.19 million deaths and millions more injuries annually. The persistently high burden has drawn attention from national and international stakeholders worldwide. Unsafe road infrastructure is one of the major risk factors for traffic safety, particularly in low- and middle-income countries.

**Methods:**

Aiming to eliminate high-risk roads in all countries, the International Road Assessment Programme (iRAP) developed a robust and evidence-based approach to support country transportation agencies.

**Results:**

Thus far, the iRAP protocols have been used to collect 1.8 million kilometers of Crash Risk Mapping and 1.5 million kilometers of Star Rating and FSI estimations in 128 countries. Deploying an observational before-and-after (or pre-post) study design, this report estimated the fatal and series injuries (FSI) saved through use of the iRAP protocols. The study is based on 441,753 kilometers of assessed roads from 1,039 projects in 74 countries. Our results show that the implementation of iRAP’s proposed countermeasures saves about 159,936 FSI annually. Throughout the lifetime of the implemented countermeasures, a total of 3.2 million FSI could be saved.

**Conclusion:**

While quantifying the success of the iRAP protocols, our results suggest an opportunity to save many millions more lives on the roads through expanding iRAP implementation to more regions and countries.

## Background

According to the World Health Organization, road traffic crashes cause 1.19 million deaths and millions more injuries every year [1]. A global macroeconomic model estimated that traffic crashes would cost the world economy US$1.8 trillion in 2015–2030, which is equivalent to an annual tax of 0.12% on global GDP (gross domestic product) [2]. Unfortunately, the level and rate of road traffic crashes have remained relatively constant over the past two decades at the global level, and even increased in some low- and middle-income countries and among children and adolescents [3].

The tremendous and persistent burden has drawn growing interest from researchers and policymakers [4]. The United Nations General Assembly declared the Decade of Action for Road Safety 2021–2030, with unanimous support from governments around the world. It specifies an explicit target of reducing road traffic deaths and injuries by at least 50% during the decade. That expands on the previous version of the Decade of Action, which included safe road infrastructure as one of the five pillars of the global plan. Due to its broad consequences beyond public health, road traffic crashes stand out as one of the most serious global threats to sustainable development. Improving road safety may help to meet multiple United National SDG (Sustainable Development Goals) targets, including halving the number of global deaths and injuries (Target 3.6), investing in infrastructure to create growth and jobs (Target 9.1), and ensuring that transport is safe and sustainable (Target 11.2) [5].

Unsafe road infrastructure is a major risk factor for traffic safety, particularly in low- and middle-income countries [6]. Existing road infrastructure is designed mainly for motorized transport, largely neglecting the safety of pedestrians, cyclists, and motorcyclists [7]. As a result, those vulnerable road users account for nearly half of the deaths and injuries on the roads.

The International Road Assessment Programme (iRAP) aims to eliminate high-risk roads through a robust and evidence-based approach [8]. The iRAP Safer Roads Investment Plan (SRIP) draw on data underpinning the iRAP Star Ratings and FSI Estimates to determine the most cost-effective road upgrades and prevent deaths and serious injuries. They provide an optimized investment of likely safety countermeasures and the business case for that investment. Each SRIP evaluates the validity and impact of a set of 94 proven road safety countermeasures. The urban-enhanced pack incorporates an additional 23 countermeasures specifically tailored for urban areas. As of December 2023, the iRAP protocols have been used to conduct 1.8 million kilometers of Crash Risk Mapping and 1.5 million kilometers of Star Rating in 128 countries, influencing over $100 billion USD of road updates [9]. It has also built a comprehensive database on key metrics of road infrastructure. The Star Rating system is adopted by the WHO to monitor global road safety performance targets [10]. That includes Target 3 (by 2030, all new roads achieve technical standards for all road users that take into account road safety, or meet a three star rating or better) and Target 4 (by 2030, more than 75% of travel on existing roads is on roads that meet technical standards for all road users that take into account road safety).

While the effectiveness of specific interventions outlined in the iRAP protocols has been demonstrated, there is a lack of rigorous evaluation for the real-world implementation of intervention packages. An identified gap is a robust statistical model to estimate the fatal and serious injuries (FSI) saved associated with the use of the iRAP protocols. The information may help iRAP in maximizing impact and assist partners in choosing the most effective design of road infrastructure. It may also support the international community in designing the most effective interventions to achieve national and global initiatives, such as those mentioned above.

The present study aims to fill that gap through systematically reviewing the documented evidence of iRAP activities and developing a statistical model to estimate the saved FSI at national and global levels. As the first global estimates of fatal and serious injuries averted by an international program, our estimates will provide a benchmark for future assessments on the subject.

### Data

Our model uses data collected as part of the implementation process of iRAP protocols. By building on the work of Road Assessment Programmes (RAP) in high-income countries and with the expertise of leading road safety research organizations worldwide, iRAP has developed four globally-consistent protocols to assess and improve the safety of roads:

1. Risk Maps use detailed crash data to illustrate the actual number of deaths and injuries on a road network.
2. Star Ratings provide a simple and objective measure of the level of safety provided by a road’s design.
3. Safer Roads Investment Plans draw on approximately 90 proven road improvement options to generate affordable and economically sound infrastructure options for saving lives.
4. Performance Tracking enables the use of Star Ratings and Risk Maps to track road safety performance and establish policy positions.

**Fig 1:**
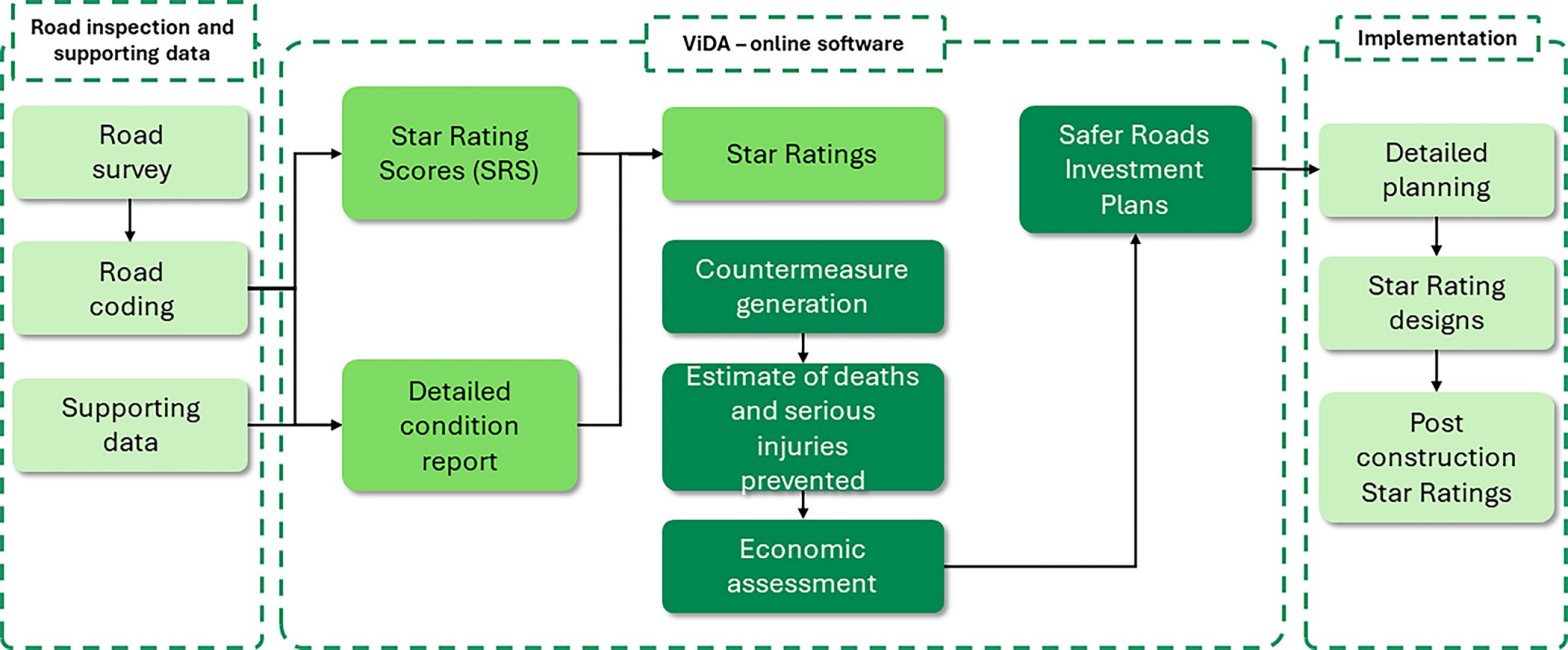
Flowchart of iRAP protocols.

The protocols specify several steps in implementing a road upgrading project. Fig 1 illustrates the process used to undertake those protocols. Overall, Star Ratings and Safer Roads Investment Plans can be used as part of a systematic, proactive approach to road infrastructure risk assessment and renewal based on research about where severe crashes are likely to occur and how they can be prevented.

The first step involves using video-based approaches to collect an extensive set of information on road infrastructure and traffic situations. The second step is to rate the safety of roads from the perspective of road users, separately for car occupants, motorcyclists, cyclists, and pedestrians. A Star Rating is given to each road segment based on known relationships between collected variables and the likelihood of crashes and their severity [11]. The risk assessment can be summarized to FSI estimates for each road segment of 100 meters. The information on road infrastructure and resulting ratings form the baseline for our estimation of saved FSI.

Based on the survey results and safety rating, the third step is to recommend network-wide countermeasures to local stakeholders and funding bodies. The actual adoption and implementation of recommended countermeasures depend on various factors (e.g., resource availability and local prioritization) and therefore may vary significantly across projects.

After local agencies partially or fully implement the recommended countermeasures, iRAP protocols recommend re-surveying the roads, re-rating road safety, and re-estimating the FSI. The updated information on road infrastructure and safety ratings forms the endline of our estimation of saved FSI.

Finally, the difference between baseline and endline FSI levels is used to measure the impacts of the iRAP protocols. Similar observational before-and-after (or pre-post) study designs have been extensively used in social, medical, and public health studies [12–14].

Like the heterogeneity with countermeasure implementation, data availability also varies significantly across projects. For countries and projects with documented infrastructure before and after countermeasure implementation, the changes in estimated FSI are calculated to measure the impacts of iRAP protocols. Dividing the total FSI reduction by the total length of assessed roads gives the average FSI reduction per kilometer of assessed roads. This average is then applied to projects without before-after comparisons.

This report uses information before and after countermeasure implementation for 134 projects in three countries: India, Mexico, and El Salvador. Then the estimated FSI reduction rate is applied to other projects to approximate the impact of iRAP protocols at the global level.

## Methods

The estimation steps for India are described in detail below. There are 22 iRAP projects in India for which implementation information is available, including the length of upgraded roads, baseline FSI (before countermeasure implementation), design FSI or post-construction FSI (after countermeasure implementation). All those projects belong to the Bloomberg Philanthropies Initiative for Global Road Safety (BIGRS). The difference between baseline and design/post-construction FSI is calculated separately for each project. The estimated total FSI saving is 33,797 for the 16,054 kilometers of assessed roads in the 22 projects. That indicates an FSI saving of 2.11 per kilometer of assessed roads.

India also has 35 iRAP projects besides BIGRS. In total, those projects assessed 7,314 kilometers of roads. Given that estimates of design/post-construction FSI are unavailable for those 35 projects, we employ an extrapolation method. This involves assuming the average FSI savings derived from the projects for which complete information is available. The extrapolation suggests a total saving of 15,433 FSI for those non-BIGRS projects. The justification for the extrapolation approach stems from the fact that all iRAP projects, whether part of the BIGRS initiative or not, adhere to the same iRAP protocols. Consequently, it is logical to assume that the impact of these projects on enhancing road safety is consistent across the board. In sum, those 57 iRAP projects in India assessed 23,368 kilometers of roads, implying an annual saving of 49,230 FSI.

The calculations steps are similar for the 75 projects in Mexico and the two projects in El Salvador. Finally, applying the FSI saving rate derived from those three countries to the 905 projects in the other 71 countries gives the extrapolated global FSI saving.

## Results

The study covers 1,039 projects in 74 countries. In total, those projects assessed 441,753 kilometers of roads (Table 1). The implemented countermeasures are estimated to save 159,936 FSI annually. On average, assessing one kilometer of roads is associated with an annual saving of 0.36 FSI per year.

**Table 1:** FSI saved by iRAP protocols in 74 countries.

Country	Annual FSI Saved	Total Length (km)
India	52,246	24,466
Pakistan	17,805	10,666
Brazil	13,291	38,506
Mexico	8,577	75,495
Philippines	8,319	8,026
Nepal	7,157	2,338
Bangladesh	5,602	1,624
Papua New Guinea	5,411	3,660
South Africa	5,068	9,502
Saudi Arabia	4,284	16,759
Tanzania, United Republic of	3,643	4,474
Lebanon	3,219	6,050
Kazakhstan	2,785	6,122
Australia	2,354	77,904
Colombia	2,122	11,212
Cambodia	1,755	558
China	1,376	2,742
Senegal	1,163	392
Egypt	1,155	3,273
Greece	807	4,897
Moldova, Republic of	766	2,604
Bosnia and Herzegovina	626	2,804
Chile	598	4,581
Dominican Republic	576	304
United Kingdom	540	14,236
Viet Nam	499	546
Indonesia	458	1,459
Thailand	455	469
Bulgaria	454	691
Ukraine	433	1,666
Netherlands	428	8,109
Hungary	393	3,924
United States	376	19,334
Slovakia	324	2,815
Ethiopia	301	529
Romania	300	746
Uruguay	254	3,587
Slovenia	252	4,065
Croatia	248	3,181
Korea, Republic of	246	1,016
Georgia	233	721
Tunisia	221	415
Côte d’Ivoire	214	65
Nicaragua	212	335
Albania	208	603
Macedonia, the former Yugoslav Republic of	195	4,632
Qatar	193	2,009
Bhutan	184	347
Peru	178	1,005
Montenegro	158	2,421
Portugal	149	5,074
Panama	135	494
Spain	133	19,675
El Salvador	128	6
Fiji	113	577
Ghana	112	110
Saint Lucia	82	293
New Zealand	81	5,042
Ecuador	70	433
Italy	60	1,052
France	56	3,980
Argentina	37	564
Dominica	25	274
Barbados	22	519
Cayman Islands	17	205
Iceland	15	4,201
Andorra	13	78
Brunei Darussalam	12	535
Cyprus	7	147
Cameroon	4	29
Puerto Rico	3	195
Samoa	2	20
Malaysia	-	368
Kenya	-	2
**TOTAL**	**159,936**	**441,753**

The top five beneficiary countries include India, Pakistan, Brazil, Mexico, and the Philippines, which combined account for about 100,328 FSI savings, or nearly two-thirds of the global FSI reduction.

**Fig 2:**
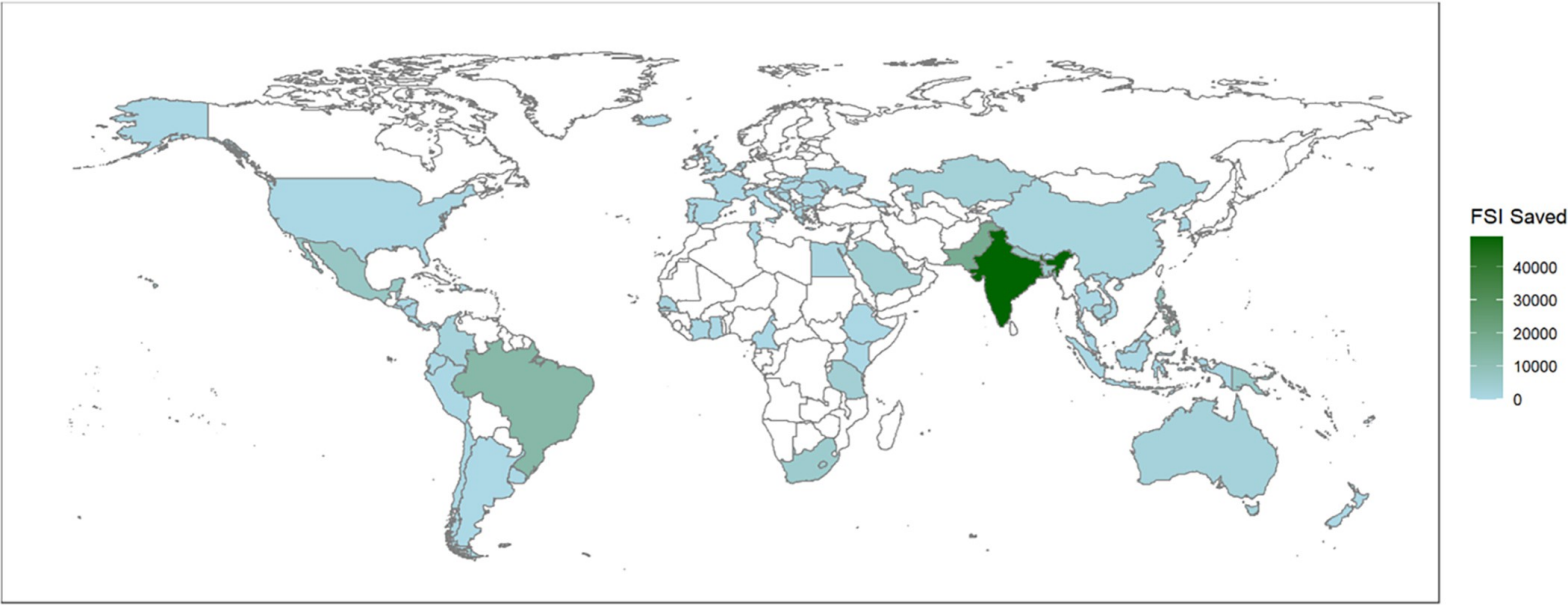
Global distribution of FSI saved by iRAP protocols. Note: the figure was generated using the R package ’maps,’ with the base layer sourced from Natural Earth.

Fig 2 presents a geographic illustration of global FSI saving. The 74 countries included in the study cover a large proportion of geographic areas in North America, Latin American and the Caribbean, Asia, Europe, and some parts of Africa. The geographical under-coverage of Africa may have implications for future applications of iRAP protocols. With a quickly growing population, fast motorization, and massive road construction, Africa urgently needs technical support on road infrastructure design and upgrading. It also creates an opportunity for the iRAP protocols to facilitate saving more FSI at the global level.

Based on the cross-sectional impacts presented in Table 1, we further estimate the lifetime savings over the life course of the road infrastructure projects. Different infrastructure countermeasures have different lifetimes. Since accurate project-level information is not available, we assume an average lifetime of 20 years for countermeasures. For projects still under construction, we assume it takes three years for them to complete and start preventing crashes and injuries.

Table 2 presents the annual and cumulative FSI savings across all projects. For previous and existing projects, the annual FSI saved gradually starts at 14,366 in 2016, gradually increases to a peak of 159,936 in 2025, stays at that level for over a decade, and eventually drops to 2,077 in 2044 as the projects included in this study reach the end of their life span. Overall, those road infrastructure renovations are projected to save more than 3 million FSI by 2044.

**Table 2:** Annual and cumulative FSI saved in the 74 countries.

Year	Annual FSI Saved	Cumulative FSI Saved
2016	14,366	14,366
2017	43,121	57,488
2018	54,131	111,619
2019	59,086	170,705
2020	67,044	237,748
2021	77,150	314,898
2022	97,352	412,250
2023	129,660	541,910
2024	157,859	699,768
2025	159,936	859,704
2026	159,936	1,019,640
2027	159,936	1,179,576
2028	159,936	1,339,512
2029	159,936	1,499,447
2030	159,936	1,659,383
2031	159,936	1,819,319
2032	159,936	1,979,255
2033	159,936	2,139,191
2034	159,936	2,299,126
2035	159,936	2,459,062
2036	145,569	2,604,632
2037	116,815	2,721,446
2038	105,804	2,827,251
2039	100,850	2,928,101
2040	92,892	3,020,993
2041	82,786	3,103,779
2042	62,584	3,166,363
2043	30,276	3,196,639
2044	2,077	3,198,716

It is worth noting that the results presented in this study may substantially underestimate the impacts of the iRAP protocols. Due to limited data availability, many projects are excluded from the present study. The analysis is only based on projects with the required information, particularly the length of assessed roads and accurately calibrated FSI estimations. On an aggregated level, our estimation only accounts for less than one-third of the 1.5 million kilometers of Star Rated roads from all iRAP projects. But there is also a possibility of overestimation among the projects included in the study. Due to the mechanism for safety assessment and FSI estimation, many of iRAP’s recommended countermeasures likely improve mobility and increase traffic volumes, which may offset some of the safety benefits from improved road infrastructure.

## Discussion

Through systematic assessments and evidence-based recommendations, iRAP has substantially improved road safety in 74 countries. Those projects represent a variety of settings in terms of socio-economic development, motorization, and road infrastructure. The demonstrated success of iRAP protocols in those heterogeneous contexts implies the potential for their applications to more places around the world. The annual saving of 159,936 FSI, which may be a gross underestimation of iRAP’s full impacts, substantially contributes to the achievement of SDG goals on global road safety.

Despite our extensive effort to calibrate and improve the estimation approach, the study is not without limitations. First, the estimation methodology involves massive extrapolation. The average FSI reduction per kilometer is estimated from the 91,810.9 kilometers of assessed roads in three countries. That only accounts for about 20% of all roads assessed across the 74 countries. Due to the tremendous heterogeneity in existing road infrastructure and countermeasure implementation, the actual FSI reduction rate may vary across projects. Second, for an observational study design, the results may be affected by other factors that are not captured in the dataset. Neither project site selection nor countermeasure implementation is random, but rather depends on local sources and prioritization. Such a non-random selection process may introduce biases in the estimates.

The study has important implications for both iRAP protocol implementation and broadly for global traffic injury prevention. For further impact evaluation, it is recommended that iRAP and local teams document project implementation in detail. That enables more actual before-after comparison, like in the cases of India, Mexico, and El Salvador in this study. Whenever possible, the teams should also collect actual traffic outcome data, such as traffic crashes, severity, injuries, and fatalities. That information not only allows a direct calculation of the protocols’ impact, but also can be used to calibrate the estimation models. The calibrated models, in turn, will help to improve the estimation accuracy for settings without actual outcome measures.

## Conclusion

As one of the five pillars in the Global Plan for the Decade of Action for Road Safety, safer roads can indeed contribute to dramatic savings of lives and injuries [15]. Our results call for more extensive applications of iRAP protocols around the world, particularly in low-and middle-income countries. As specified in the protocols, activities may include improving safety-conscious planning, design, construction, and renovation. It also includes regularly assessing roads for safety and encouraging authorities to consider all transportation modes to meet people’s needs for safe mobility. Such a comprehensive package has the potential to substantially reduce the likelihood of traffic crashes and severity, and save millions of lives and injuries around the world.
